# Budd-Chiari Syndrome Caused by Polycythemia Vera: A Case Report

**DOI:** 10.7759/cureus.45527

**Published:** 2023-09-19

**Authors:** Joana Camões Neves, Filipa Rodrigues, Isabel Apolinário, Marina Alves, Olinda Sousa Caetano

**Affiliations:** 1 Gastroenterology, Hospital de Braga, Braga, PRT; 2 Internal Medicine, Hospital de Braga, Braga, PRT

**Keywords:** case report, anticoagulation, jak2 mutation, hepatic vein thrombosis, liver diseases, polycythemia vera, budd-chiari syndrome

## Abstract

Budd-Chiari syndrome (BCS) is a rare condition characterized by the obstruction of hepatic venous outflow. It has various potential etiologies, with myeloproliferative neoplasms representing the most prevalent pathogenic association. Here, we present the case of a 51-year-old male who manifested abdominal pain and ascites. Subsequent clinical investigation revealed the presence of BCS secondary to a myeloproliferative syndrome, specifically polycythemia vera. This case emphasizes the importance of diagnosing BCS and conducting a thorough investigation into its underlying etiology.

## Introduction

Budd-Chiari syndrome (BCS) is an uncommon but clinically challenging condition [1] characterized by the obstruction of venous outflow from the suprahepatic veins to the cavoatrial junction [2]. It can be classified as primary, resulting from intraluminal venous injury, or secondary, occurring due to compression or extrinsic invasion [3]. The primary form represents the most frequent presentation, with an estimated prevalence of one case per million people per year. The average age of diagnosis is between 35 and 40 years [1,4]. It manifests in several ways, with abdominal pain, ascites, and hepatomegaly being the most common symptoms. In fact, patients can present from asymptomatic forms to the appearance of complications of liver disease [5].

After diagnosing BCS, an underlying thrombotic disorder is identified in 75% of patients [1]. Studies suggest that in about one-third of cases, more than one condition is identified [1,3]. Myeloproliferative neoplasms (MPNs) are the leading cause of BCS [6], with a high prevalence of the *JAK2V617F* mutation in this syndrome. Among the associated MPNs, polycythemia vera (PV) is the most frequent, corresponding to about half of the cases [7].

We report the case of a 51-year-old male diagnosed with BCS secondary to PV. This case highlights the significance of assessing prothrombotic conditions during the investigation of BCS, as well as emphasizes the importance of early diagnosis and prompt treatment.

## Case presentation

A 51-year-old male, independent in activities of daily living and working as an education assistant, was admitted to the emergency department at Braga Hospital, in Portugal, in January 2023. He had mesogastric pain, abdominal distension, and early satiety after one month of evolution. On physical examination, he was oriented, hemodynamically stable, apyretic, with ascites and worsening pain on palpation of the right upper quadrant of the abdomen. He presented a personal history of polymyalgia rheumatica in remission, treated with prednisolone 7.5 mg/day for one year, with no identified adverse effects. He had no smoking or alcohol habits.

Laboratory data indicated normal levels of hemoglobin and platelets, a slight increase in hematocrit, mild leukocytosis, increased transaminases and cholestasis parameters, mild hyperbilirubinemia, coagulation parameters within the normal range, and albumin at the lower limit of normal with a normal total protein value (Table 1).

**Table 1:** Laboratory data. Hgb: hemoglobin; WBC: white blood cells; AST: aspartate aminotransferase; ALT: alanine aminotransferase; PT: prothrombin time; PTT: partial thromboplastin time; INR: international normalized ratio; LDH: lactate dehydrogenase; CRP: C-reactive protein

Test	Result	Normal range
Hgb (g/dL)	16.5	13.5–17.0
Hematocrit (%)	51.2	40.0–49.5
WBC (×10^3^/µL)	14.2	4.0–11.0
Platelets (×10^3^/µL)	335	150–400
Sodium (mmol/L)	142	136–145
Potassium (mmol/L)	4.3	3.5–5.1
Chloride (mmol/L)	104	98–107
Urea (mg/dL)	24	19–49
Creatinine (mg/dL)	1.0	0.6–1.2
Glucose (mg/dL)	73	70–110
Total proteins (g/dL)	5.7	5.7–8.2
Albumin (g/dL)	3.4	3.4–5.0
AST (U/ L)	87	12–40
ALT (U/ L)	190	7–40
Total bilirubin (mg/dL)	1.76	0.30–1.20
Direct bilirubin (mg/dL)	1.03	<0.30
Alkaline phosphatase (U/L)	317	46–116
Gamma-glutamyltransferase (U/L)	776	<73
PT (seconds)	13	8–14
INR	1.11	0.80–1.20
PTT (seconds)	36.4	25.0–37.0
LDH (U/L)	269	120–246
CRP (mg/L)	3.90	<5.00
Procalcitonin (ng/mL)	0.05	<0.05

**Figure 1:**
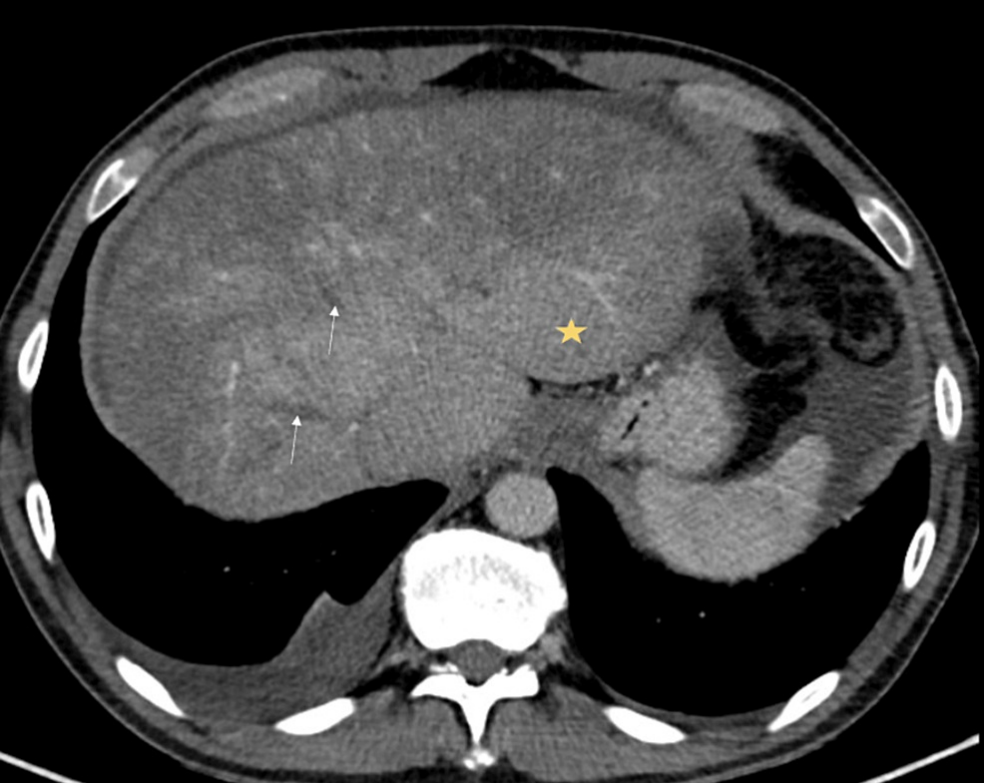
Contrast-enhanced abdominal CT in venous phase demonstrating findings suggestive of Budd-Chiari syndrome. The CT shows a heterogeneous pattern in the contrast uptake of the liver, with early uptake of the caudate lobe (marked with yellow star) associated with the absence of uptake of the suprahepatic veins (marked with white arrows).

**Figure 2:**
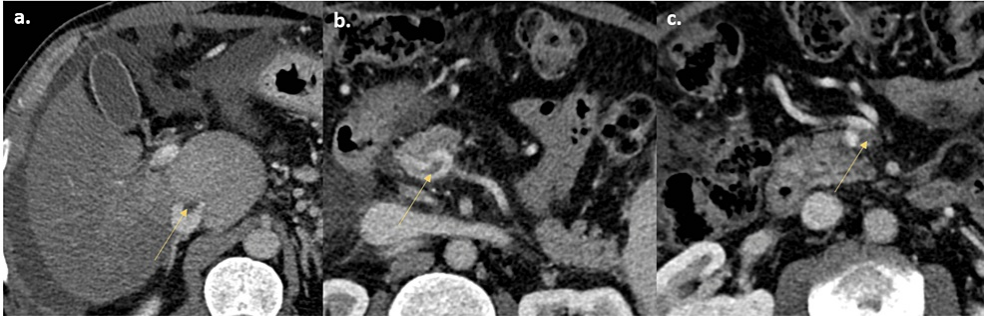
Three sections of abdominal CT with contrast in the venous phase showing venous thrombosis. Venous thrombi are visualized in image (a) in the inferior vena cava (arrow), in image (b) in the splenoportal venous confluence (arrow), and in image (c) in the superior mesenteric vein (arrow).

An abdominopelvic computed tomography (CT) scan revealed moderate ascites, hepatosplenomegaly with hypertrophy of the caudate lobe, and alterations in the pattern of liver perfusion, with an earlier and more homogeneous uptake of the caudate lobe and heterogeneous uptake of the cranial planes, presenting a mottled and multinodular appearance. Venous thrombosis was detected, with a small thrombus in the inferior vena cava and partial thrombosis of the superior mesenteric vein and splenoportal confluence (Figures 1, 2).

**Figure 3:**
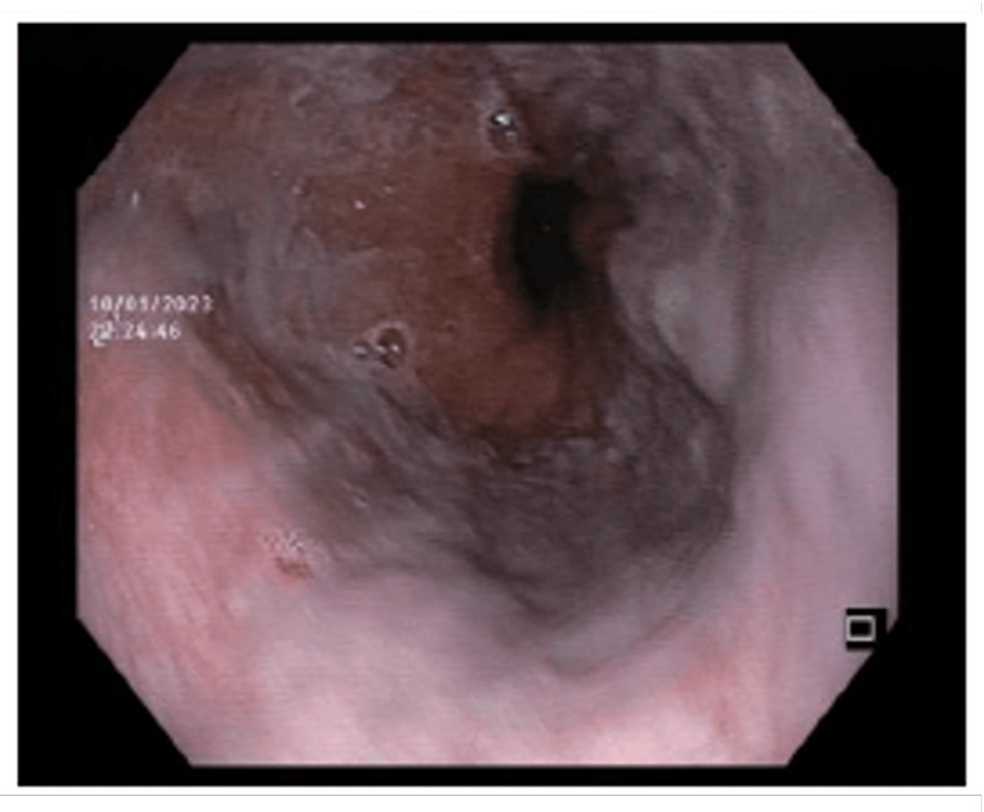
Upper digestive endoscopy showing small esophageal varices in the distal esophagus.

Upper digestive endoscopy revealed the presence of small esophageal varices in the distal esophagus that flattened out on insufflation and no signs of portal hypertensive gastropathy (Figure 3). The patient started anticoagulation and diuretic therapy and was subsequently hospitalized for further evaluation of BCS.

**Figure 4:**
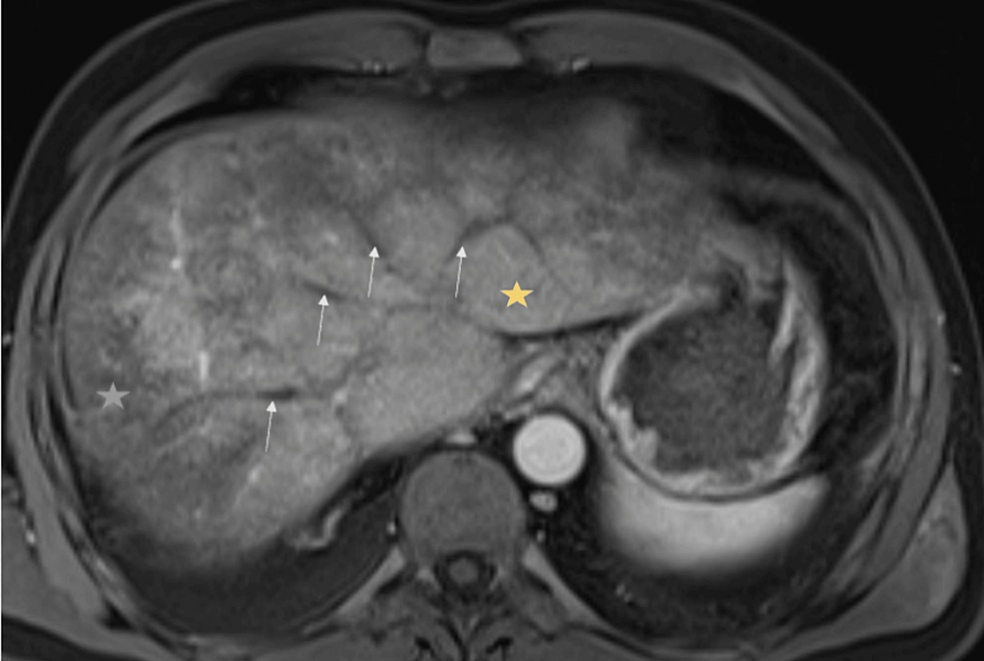
Abdominal MRI post-contrast T1-weighted sequence in venous phase demonstrating findings compatible with Budd-Chiari syndrome. The MRI shows a mottled pattern, with areas of early uptake in the caudate lobe (marked with a yellow star) that coexist with areas of more peripheral uptake (blue star), associated with the absence of opacification of the suprahepatic veins (marked with white arrows).

During hospitalization, magnetic resonance imaging was performed to characterize venous anomalies more accurately, considering invasive therapy as an option. It revealed findings consistent with the CT scan, including the presence of ascites, hypertrophy of the caudate lobe, persistent alteration in the hepatic perfusion pattern, exhibiting areas with a mottled appearance, as well as areas of regeneration. Moreover, there was an absence of enhancement in the hepatic veins following intravenous paramagnetic contrast administration, and maintenance of previously identified thrombi, with a permeable portal vein trunk (Figure 4).

A transthoracic echocardiogram and an electrocardiogram were conducted without relief alterations. An ultrasound-guided diagnostic paracentesis was performed, showing a serum ascites gradient greater than 1.1 g/dL, with no increase in polymorphonuclear cells, which supported the presence of ascites associated with portal hypertension in the context of BCS. The ascitic fluid culture yielded negative results, and cytology showed no evidence of malignant cells.

From the etiological investigation, serological tests for HIV and hepatitis A, B, and C returned negative results, and alpha-fetoprotein values were within the normal range. Colonoscopy did not reveal any malignancy or other changes. Furthermore, protein C and protein S values were normal, and tests for antiphospholipid, antinuclear, and antineutrophil cytoplasmic antibodies showed negative results. Prothrombin gene, factor V Leiden, and *MTHFR *gene mutation tests were also negative. A search for *JAK2 *mutations was performed, with a positive result.

The bone marrow biopsy revealed a hypercellular bone marrow for the patient’s age, with panmyelosis and dysplasia in the megakaryocytic line. These findings are indicative of MPN, more specifically the PV subtype.

Therefore, the diagnosis of BCS with an etiology related to PV was confirmed. The case was discussed with Gastroenterology and Interventional Radiology, considering the potential for invasive therapeutic interventions, although no intervention was currently indicated. In addition to anticoagulation, he started cytoreductive treatment with hydroxyurea and required phlebotomy.

During hospitalization, the patient remained hemodynamically stable, with controlled pain, and showed improvement in ascites and laboratory data. Subsequently, the patient was discharged and referred to a multidisciplinary group for further consultations and follow-up.

## Discussion

BCS is a potentially life-threatening condition in which hepatic venous outflow obstruction causes hepatic congestion and subsequent fibrosis. The elevated sinusoidal pressure leads to ascites, portal hypertension, and the development of collaterals [8].

In this case, we dealt with primary BCS. The presentation of BCS is highly diverse and nonspecific, highlighting the importance of a high index of suspicion. The patient in this case had abdominal pain, which is the most common symptom that leads patients with BCS to seek medical attention. Besides pain, ascites and hepatomegaly, which were present in this case, are the most frequently encountered symptoms [9].

In addition to MPN, which is the most common cause of BCS, there are other associated factors such as antiphospholipid antibody syndrome, mutations in factor V Leiden and prothrombin gene, protein C and S deficiencies, and neoplasms, among others [1]. Therefore, in the etiological assessment, it is crucial to consider the various potential causes, as was done in this patient.

After actively researching the etiology in this case, we identified the presence of the *V617F *mutation in the *JAK2 *gene, and bone marrow biopsy confirmed PV as the underlying cause. The identification of the etiology is extremely relevant to directing the therapeutic strategy. The association of MPN with BCS is underestimated. One factor supporting this is the absence of changes in peripheral blood secondary to hypersplenism frequently found in these patients [10]. Several studies have suggested that in cases of BCS secondary to MPN, a blood count with normal cellular parameters is more commonly observed [3,11,12]. This case describes PV with normal hemoglobin values, despite a slight increase in hematocrit.

Imaging examinations are a crucial component in the diagnostic approach for patients. Typical findings, such as a dysmorphic liver with an enlarged caudate lobe, the presence of thrombi, and the formation of collaterals, are highly suggestive and were observed in our patient [13].

BCS is associated with high mortality (>80% in three years) [1], underscoring the need for timely patient management to decrease complications. Treatment should focus on restoring hepatic flow and monitoring the consequences of hepatic congestion, along with managing the underlying prothrombotic condition when identified. Thus, the prompt initiation of diuretics and anticoagulant therapy is crucial, as was done in this patient. Treating the underlying cause, in this case with hydroxyurea and phlebotomy, is essential, as it has been associated with improved outcomes [1,14].

This patient had no indication for intervention at the time of diagnosis, which could potentially improve his prognosis. Endovascular therapy is associated with high technical and clinical success rates. Other options include transjugular intrahepatic portosystemic shunt or, as a last resort, liver transplantation [15].

## Conclusions

This case report highlights the association between two rare but commonly related disorders. Indeed, it is essential to diagnose BCS and conduct an exhaustive search for the associated etiology, in this case, PV. Timely treatment is crucial to restore hepatic venous flow, and in extreme cases, liver transplantation may be necessary.

## References

[1] Garcia-Pagán JC, Valla DC (2023). Primary Budd-Chiari syndrome. N Engl J Med.

[2] Metzger PB, Costa KR, Silva SL, Dos Santos VR, Nunes V, Freire MQ, Mello MO (2021). Budd-Chiari syndrome due to hepatic venous web outflow obstruction: percutaneous treatment with balloon angioplasty. J Vasc Bras.

[3] Mancuso A (2014). An update on management of Budd-Chiari syndrome. Ann Hepatol.

[4] Ollivier-Hourmand I, Allaire M, Goutte N (2018). The epidemiology of Budd-Chiari syndrome in France. Dig Liver Dis.

[5] Darwish Murad S, Plessier A, Hernandez-Guerra M (2009). Etiology, management, and outcome of the Budd-Chiari syndrome. Ann Intern Med.

[6] Smalberg JH, Arends LR, Valla DC, Kiladjian JJ, Janssen HL, Leebeek FW (2012). Myeloproliferative neoplasms in Budd-Chiari syndrome and portal vein thrombosis: a meta-analysis. Blood.

[7] Găman MA, Cozma MA, Manan MR (2023). Budd-Chiari syndrome in myeloproliferative neoplasms: a review of literature. World J Clin Oncol.

[8] Simonetto DA, Yang HY, Yin M (2015). Chronic passive venous congestion drives hepatic fibrogenesis via sinusoidal thrombosis and mechanical forces. Hepatology.

[9] Murphy PZ, Thomas J, McClelland TP (2021). Pain management of Budd Chiari syndrome in the primary care setting: a case study. Innov Pharm.

[10] Valla D, Casadevall N, Lacombe C (1985). Primary myeloproliferative disorder and hepatic vein thrombosis. A prospective study of erythroid colony formation in vitro in 20 patients with Budd-Chiari syndrome. Ann Intern Med.

[11] Westbrook RH, Lea NC, Mohamedali AM (2012). Prevalence and clinical outcomes of the 46/1 haplotype, Janus kinase 2 mutations, and ten-eleven translocation 2 mutations in Budd-Chiari syndrome and their impact on thrombotic complications post liver transplantation. Liver Transpl.

[12] Goulding C, Uttenthal B, Foroni L (2008). The JAK2(V617F) tyrosine kinase mutation identifies clinically latent myeloproliferative disorders in patients presenting with hepatic or portal vein thrombosis. Int J Lab Hematol.

[13] Brancatelli G, Vilgrain V, Federle MP, Hakime A, Lagalla R, Iannaccone R, Valla D (2007). Budd-Chiari syndrome: spectrum of imaging findings. AJR Am J Roentgenol.

[14] Hamulyák EN, Daams JG, Leebeek FW, Biemond BJ, Te Boekhorst PA, Middeldorp S, Lauw MN (2021). A systematic review of antithrombotic treatment of venous thromboembolism in patients with myeloproliferative neoplasms. Blood Adv.

[15] Mukhiya G, Zhou X, Han X, Jiao D, Pokhrel G, Li Y, Pokhrel S (2022). Evaluation of outcome from endovascular therapy for Budd-Chiari syndrome: a systematic review and meta-analysis. Sci Rep.

